# Cancer mortality 1981–2016 and contribution of specific cancers to current socioeconomic inequalities in all cancer mortality: A population-based study

**DOI:** 10.1016/j.canep.2021.102010

**Published:** 2021-10

**Authors:** Denise Brown, David I. Conway, Alex D. McMahon, Ruth Dundas, Alastair H. Leyland

**Affiliations:** a MRC/CSO Social and Public Health Sciences Unit, University of Glasgow, G3 7HR, UK; b School of Medicine, Dentistry and Nursing, University of Glasgow, G2 3JZ, UK

**Keywords:** ECIS, European Cancer Information System, IARC, International Agency for Research on Cancer, ICD, International Classification of Diseases, NHS, National Health Service, NMSC, non-melanoma skin cancer, PHS, Public Health Scotland, RII, relative index of inequality, SII, slope index of inequality, WHO, World Health Organization, Neoplasms, Mortality, Socioeconomic factors, Scotland

## Abstract

**Background:**

In many high-income countries cancer mortality rates have declined, however, socioeconomic inequalities in cancer mortality have widened over time with those in the most deprived areas bearing the greatest burden. Less is known about the contribution of specific cancers to inequalities in total cancer mortality.

**Methods:**

Using high-quality routinely collected population and mortality records we examine long-term trends in cancer mortality rates in Scotland by age group, sex, and area deprivation. We use the decomposed slope and relative indices of inequality to identify the specific cancers that contribute most to absolute and relative inequalities, respectively, in total cancer mortality.

**Results:**

Cancer mortality rates fell by 24 % for males and 10 % for females over the last 35 years; declining across all age groups except females aged 75+ where rates rose by 14 %. Lung cancer remains the most common cause of cancer death. Mortality rates of lung cancer have more than halved for males since 1981, while rates among females have almost doubled over the same period.

**Conclusion:**

Current relative inequalities in total cancer mortality are dominated by inequalities in lung cancer mortality, but with contributions from other cancer sites including liver, and head and neck (males); and breast (females), stomach and cervical (younger females). An understanding of which cancer sites contribute most to inequalities in total cancer mortality is crucial for improving cancer health and care, and for reducing preventable cancer deaths.

## Highlights

• Cancer mortality rates have declined but inequalities in rates have widened.
• Relative inequalities are dominated by inequalities in lung cancer mortality.
• There is also a contribution from liver and head and neck cancers (men).
• And from breast cancer (women), stomach and cervical cancer (younger women).
• Understanding these patterns is important in reducing preventable cancer deaths.

## 1 Introduction

Cancer is the second leading cause of death worldwide with approximately one in six deaths globally [1], and one in four deaths in Europe and in the UK, due to cancer. It is the leading cause of death in Scotland (population of approximately 5.4 million) with 16,250 cancer deaths recorded in 2018 (27 % of total deaths). Cancer mortality rates have fallen by 24 % for males and by 10 % for females since 1981; small declines compared to those observed for other major causes of death, such as ischaemic heart disease and stroke [2].

Cancer mortality rates in Scotland are higher than the UK as a whole for all cancers [3] and for several specific cancers [4]. Lung cancer mortality rates, in particular, are around a third higher than the UK average [5]. While current male lung cancer mortality rates in Scotland are comparable to European rates, rates for females in Scotland are more than twice the rates for females in Europe [6,7].

Describing the magnitude of social inequalities in cancer and monitoring progress in reducing social inequalities in cancer has been recommended as a priority for research [8]. A recent publication from the International Agency for Research on Cancer (IARC) highlights the large differences in cancer incidence, survival and mortality that exists between and within countries across social groups [9]. In Scotland, overall cancer mortality rates are currently around 74 % higher in the most deprived areas compared to the least deprived [7], reflecting persistent inequalities in cancer incidence [10] and cancer survival [11].

Reducing social inequalities in total cancer mortality requires an understanding of the contribution of site-specific cancers to absolute and relative inequalities in cancer mortality. Our work examines the extent to which specific cancers contribute to current inequalities in cancer mortality in Scotland. We also look at long-term trends in cancer mortality in Scotland by age group, sex, and area deprivation using high-quality routinely collected Scottish mortality and population records.

## 2 Methods

### 2.1 Cancer mortality

We examine cancer deaths in the four-year period around each census 1981 (1980−83), 1991 (1991−93), 2001 (2000−03) and 2011 (2010−13), and in the four-year period around 2016 (2015−18). We used four years of death data around each period to improve robustness of the mortality rates; however note that deaths in 1990 were excluded from the analysis due to an issue with assigning some deaths to postcodes at that time [12]. Individual level death data were obtained from vital events records held by National Records of Scotland. These data included information on age, sex, underlying cause of death and area of residence (used to assign deaths to an area deprivation measure). A small percentage of cancer deaths (around 0.6 %) were excluded from the analysis due to insufficient information on age, sex or area of residence. The underlying cause of death was coded in accordance with the International Classification of Diseases (ICD) using ICD-9 codes 140-208 for cancer deaths in 1981 and 1991 and ICD-10 codes C00-C97 for cancer deaths in 2001, 2011 and 2016.

### 2.2 Specific cancers mortality

We examined deaths for all cancers and for the most common cancer sites in males and females in Scotland in 2018 as detailed in the Scottish Cancer Registry, Public Health Scotland (PHS) [13] or, for comparison, in Europe based on the European Cancer Information System (ECIS) [6] (Supplementary Appendix Table A.1).

### 2.3 Population size

We use census population estimates of the usually resident population on census day in Scotland in 1981 (n = 5,178,248), 1991 (n = 5,106,135), 2001 (n = 5,062,011), 2011 (n = 5,295,403), and mid-year small area population estimates [14] for 2016 (n = 5,404,700).

### 2.4 Area deprivation

Carstairs deprivation scores were used to assess area-level deprivation. Scores were created, following the 1981, 1991, 2001 and 2011 censuses [15], by combining four variables taken from census data; male unemployment (proportion of economically active males seeking or waiting to start work), low social class (proportion of people living in households with the household reference person in semi-skilled or unskilled manual occupations), overcrowding (proportion of people living in households at a density of more than one person per room) and no car ownership (proportion of people living in households with no car). Carstairs scores, historically, were created at the postcode sector level of geography (average population size 5,233, min: 52, max: 21,159 in 2011) but were created for additional census geography in 2011, including 2011 data zones (average population size 759, min: 147, max: 2901) [12]. Postcode sectors, available from death certificates, were linked to the appropriate version of Carstairs (i.e. 1981 Carstairs scores assigned to deaths in 1981). For deaths in 2016, mid-year estimates of the population (for calculation of directly age-standardised mortality rates) were not available at postcode sector level. Deaths in 2016 (and 2011 for comparison) were assigned to 2011 Carstairs scores based on 2011 data zones, for which mid-year estimates of the population are available. Information on area of residence from death certificates in 2011 and 2016 was first linked to 2011 census output areas, from which 2011 data zones are built, and then to 2011 data zones.

### 2.5 Analyses

Cancer mortality rates were age-standardised using the 2013 European standard population. We present rates by 15-year age groups for all cancers and for age groups 30−44, 45−59, 60−74 and 75+ for specific cancers, due to small numbers of deaths at younger ages. Cancer mortality rates shown by Carstairs scores divided into population-weighted deprivation fifths are for all ages.

The slope index of inequality (SII) and relative index of inequality (RII) are used to examine current absolute and relative inequalities [16]. Different methods have been proposed for the calculation of SII and RII [17]. We calculate SII here by fitting a linear regression [18] of the age-standardised rates, weighted by the size of the deprivation groups. The larger the slope coefficient the higher the impact of deprivation on absolute differences in rates. The SII can be calculated for all cancers or for specific cancers, with the sum of the SII for specific cancers equal to the SII for all cancers. This allows us to decompose the SII and examine the contribution of specific cancers to inequalities in all cancer mortality. The RII is obtained by dividing the SII by the population mean rate and can be decomposed in the same way. An RII value of zero indicates that there is no inequality while a value of one suggests that cancer mortality rates in the most deprived areas are about 50 % above the average cancer mortality rate (and about 50 % lower than average in the least deprived areas).

## 3 Results

### 3.1 Cancer mortality

There were 13,923, 15,054, 14,968, 15,575 and 15,945 cancer deaths, respectively, on average per year over each period (Supplementary Appendix Table A.2), accounting for 22.0 %, 24.6 %, 26.1 %, 28.8 % and 28.2 %, respectively, of the average number of all deaths per year in each period. Male age-standardised cancer mortality rates fell from 497 to 379 per 100,000 population between 1981 and 2016, a reduction of 24 % (Table 1). Over the same period, female rates decreased by 10 %, from 297 to 267 per 100,000 population. Most age groups saw a reduction in rates over the last 35 years. The exception is for females, aged 75+, where rates increased by 14 %. Corresponding mortality rates for all cancers, excluding non-melanoma skin cancer (NMSC), are shown in Supplementary Appendix Table A.3.

**Table 1:** Age-standardised all cancers and specific cancers mortality rates (per 100,000 population) for males and females, 1981 (1980-83), 1991 (1991-93), 2001 (2000-03), 2011 (2010-13) and 2016 (2015-18). Rates are shown for 15-year age groups (for cancer sites, rates are shown for 15-year age groups from 30+). Rates for all ages are shown in bold-italics.

	Males						Females					
	Years					*% Change*	Years					*% Change*
Age	1981	1991	2001	2011	2016	***1981−2016***	1981	1991	2001	2011	2016	***1981−2016***
***All cancers (ICD-9 140–208; ICD-10 C00–C97)***												
0−14	5	3	4	2	2	***−57***	4	4	2	2	2	–
15−29	9	6	6	5	4	***−51***	7	6	5	5	4	***−45***
30−44	37	31	24	21	22	***−42***	53	43	33	30	29	***−46***
45−59	280	256	206	160	148	***−47***	252	236	194	163	148	***−41***
60−74	1181	1199	1029	838	766	***−35***	679	746	675	620	563	***−17***
75+	2603	2795	2689	2563	2406	***−8***	1339	1442	1543	1553	1522	***14***
***All ages***	***497***	***510***	***461***	***407***	***379***	***−24***	***297***	***312***	***298***	***283***	***267***	***−10***
***Lung (ICD-9 162; ICD-10 C33-34)***												
30−44	8	5	3	3	3	***−66***	4	4	3	2	2	–
45−59	120	85	58	41	35	***−70***	51	45	40	38	30	***−41***
60−74	529	481	343	257	217	***−59***	146	205	196	201	177	***21***
75+	855	822	683	605	520	***−39***	137	229	311	372	371	***170***
***All ages***	***191***	***172***	***131***	***106***	***90***	***−53***	***48***	***64***	***69***	***75***	***69***	***45***
***Colorectal (ICD-9 153, 154.0−154.1; ICD-10 C18−20)***												
30−44	3	3	2	2	3	–	3	2	2	2	3	–
45−59	27	31	21	15	16	***−41***	21	21	14	13	14	***−35***
60−74	115	122	111	83	76	***−34***	85	80	57	49	45	***−47***
75+	349	354	311	285	266	***−24***	265	220	191	171	170	***−36***
***All ages***	***57***	***59***	***51***	***43***	***40***	***−29***	***43***	***38***	***30***	***27***	***26***	***−39***
***Prostate (ICD-9 185; ICD-10 C61)***												
30−44	0	0	0	0	0	–						
45−59	5	5	6	4	4	***−2***						
60−74	66	83	79	63	63	***−5***						
75+	332	449	458	441	398	***20***						
***All ages***	***42***	***55***	***55***	***51***	***47***	***13***						
***Breast (female) (ICD-9 174; ICD-10 C50)***												
30−44							19	15	11	9	9	***−53***
45−59							74	68	51	38	32	***−57***
60−74							111	114	91	74	64	***−42***
75+							186	209	193	177	166	***−11***
***All ages***							***54***	***55***	***45***	***38***	***34***	***−37***
***Oesophagus (ICD-9 150; ICD-10 C15)***												
30−44	2	2	1	1	1	–	1	0	0	0	0	–
45−59	14	19	19	16	14	***4***	5	7	5	4	4	***−23***
60−74	45	62	63	64	61	***34***	25	29	23	21	19	***−23***
75+	99	110	132	132	126	***27***	62	77	73	67	61	***−3***
***All ages***	***20***	***24***	***27***	***26***	***24***	***25***	***11***	***13***	***11***	***10***	***9***	***−13***
***Pancreas (ICD-9 157; ICD-10 C25)***												
30−44	2	1	1	1	1	–	1	1	1	1	0	–
45−59	12	10	11	9	8	***−28***	8	6	6	6	5	***−36***
60−74	48	44	40	42	42	***−12***	32	32	30	30	31	***−4***
75+	106	101	81	94	90	***−16***	83	72	72	75	76	***−8***
***All ages***	***20***	***19***	***16***	***17***	***17***	***−16***	***15***	***13***	***13***	***13***	***13***	***−11***
***Liver (ICD-9 155; ICD-10 C22)***												
30−44	1	1	1	1	1	–	0	1	0	0	0	–
45−59	4	5	5	7	8	***135***	2	2	3	3	4	–
60−74	14	17	23	32	36	***152***	6	7	11	14	17	***194***
75+	25	37	39	70	94	***269***	11	18	24	35	45	***317***
***All ages***	***5***	***7***	***8***	***13***	***16***	***196***	***2***	***3***	***5***	***6***	***8***	***225***
***Bladder (ICD-9 188; ICD-10 C67)***												
30−44	0	0	0	0	1	–	0	0	0	0	0	–
45−59	8	7	4	3	2	***−71***	3	2	2	2	2	–
60−74	46	41	33	24	24	***−49***	18	16	12	11	9	***−49***
75+	144	156	130	133	123	***−15***	44	48	47	49	46	***5***
***All ages***	***22***	***22***	***18***	***17***	***16***	***−30***	***8***	***7***	***7***	***7***	***6***	***−19***
***Head and neck (ICD-9 140−149, 160−161; ICD-10 C11−14, C30−32)***												
30−44	1	2	1	1	1	–	1	1	0	0	1	–
45−59	9	14	14	12	9	***9***	4	4	4	4	4	–
60−74	28	41	37	37	40	***44***	10	11	11	12	13	***28***
75+	66	52	54	54	63	***−6***	22	20	17	21	22	***0***
***All ages***	***13***	***15***	***14***	***13***	***14***	***13***	***5***	***5***	***4***	***5***	***5***	***0***
***Stomach (ICD-9 151; ICD-10 C16)***												
30−44	3	2	1	1	1	–	2	1	1	1	1	–
45−59	25	14	9	6	5	***−78***	9	6	4	3	3	***−66***
60−74	98	75	50	29	22	***−77***	43	31	20	12	9	***−80***
75+	196	168	133	102	80	***−59***	137	88	68	47	38	***−72***
***All ages***	***39***	***31***	***22***	***15***	***12***	***−69***	***22***	***14***	***10***	***7***	***6***	***−74***
***Non-Hodgkin lymphoma (ICD-9 200, 202; ICD-10 C82−86)***												
30−44	2	2	1	1	1	–	1	1	1	1	0	–
45−59	5	8	6	4	4	***−18***	4	6	5	3	2	***−48***
60−74	20	22	27	20	18	***−9***	16	21	19	14	14	***−15***
75+	35	57	60	60	67	***93***	27	40	48	46	45	***69***
***All ages***	***8***	***11***	***11***	***10***	***10***	***27***	***6***	***9***	***9***	***7***	***7***	***10***
***Kidney (ICD-9 189.0; ICD-10 C64)***												
30−44	1	1	1	1	1	–	0	1	0	0	0	–
45−59	7	8	7	6	6	***−14***	3	4	3	2	3	–
60−74	20	23	24	***20***	20	***−3***	11	12	12	11	10	***−12***
75+	36	41	50	55	62	***71***	19	21	25	29	32	***70***
***All ages***	***8***	***9***	***10***	***9***	***10***	***23***	***4***	***5***	***5***	***5***	***5***	***19***
***Leukaemia (ICD-9 204−208; ICD-10 C91−95)***												
30−44	2	2	2	1	1	–	2	2	1	1	1	–
45−59	6	4	4	3	3	***−52***	3	3	3	2	2	–
60−74	19	20	22	22	20	***4***	11	11	12	10	9	***−14***
75+	61	49	67	77	69	***12***	33	28	35	39	37	***11***
***All ages***	***11***	***9***	***11***	***12***	***10***	***−4***	***6***	***6***	***6***	***6***	***6***	***−11***
***Ovary (ICD-9 183.0; ICD-10 C56)***												
30−44							4	2	1	2	1	–
45−59							19	20	14	10	9	***−52***
60−74							40	42	43	37	31	***−23***
75+							43	55	67	65	65	***51***
***All ages***							***15***	***16***	***16***	***14***	***13***	***−14***
***Corpus uteri (ICD-9 182; ICD-10 C54)***												
30−44							0	0	0	0	0	–
45−59							3	2	3	2	3	–
60−74							11	12	11	14	16	***42***
75+							20	18	24	28	34	***69***
***All ages***							***4***	***4***	***5***	***5***	***6***	***44***
***Cervix uteri (ICD-9 180; ICD-10 C53)***												
30−44							6	5	3	3	3	***−38***
45−59							11	9	6	5	5	***−57***
60−74							20	16	8	6	5	***−73***
75+							22	17	13	10	10	***−54***
***All ages***							***9***	***7***	***4***	***4***	***4***	***−59***

Cancer mortality rates are rounded to the nearest whole number while % change shows the percentage change in actual (unrounded) rates. Note that % change is not calculated, for a particular age group, when rates are consistently <5 per 100,000 population over time.

### 3.2 Specific cancers mortality

Mortality rates for specific cancers are presented by age group in Table 1. Lung cancer remains the most common cause of cancer death for males, despite rates having more than halved since 1981. It is also the most common cause of cancer death in females with rates almost doubling over the last 35 years. Increases in female lung cancer mortality were mainly driven by increases in mortality among those aged 75 and older; however there was also an increase in 60−74 year olds.

Over the last 35 years female breast cancer mortality rates have declined by around a third, with reductions in mortality observed across all age groups. There have also been large reductions for females, across all age groups, in deaths from colorectal cancer (39 %), stomach cancer (74 %) and cervical cancer (59 %). Liver cancer deaths have increased four-fold. Males also saw large reductions in mortality rates from colorectal cancer (29 %) and stomach cancer (69 %) as well as bladder cancer (30 %). Deaths from cancer of the oesophagus have risen by a quarter over the period, with liver cancer deaths now three times as high as in 1981.

### 3.3 Cancer mortality by area deprivation

Cancer mortality rates are shown by deprivation fifths for males (Table 2a) and females (Table 2b) separately. Socioeconomic inequalities in cancer mortality assessed at 2011 data zone level are wider than when assessed at postcode sector level (Supplementary Appendix Figure A.1). This is likely due to areas being more homogenous in 2011 data zones, which have smaller population sizes on average. As a result we have examined change over time between 1981 and 2011 (postcode sectors), 2011 and 2016 (2011 data zones) and have approximated the overall change between 1981 and 2016.

**Table 2a:** Age-standardised all cancer and specific cancers mortality rates (per 100,000 population) for males, all ages, in 1981 (1980-83), 1991 (1991-93), 2001 (2000-03), 2011 (2010-13) and 2016 (2015-18) by most to least deprived fifth. Rates for all Scotland are shown in bold-italics.

	Years						% Change		
	1981^a^	1991^a^	2001^a^	2011^a^	2011^b^	2016^b^	***1981−2011***^a^	***2011−2016***^b^	***1981−2016***^c^
***All cancers (ICD-9 140−208; ICD-10 C00–C97)***									
Most deprived	611	622	555	516	537	504	−15	−6	−21
2	534	544	490	441	452	431	−17	−5	−21
3	492	503	461	397	400	377	−19	−6	−24
4	438	470	427	368	352	334	−16	−5	−20
Least deprived	430	417	381	344	318	296	−20	−7	−26
***All Scotland***	***497***	***510***	***461***	***407***	***407***	***379***	***−18***	***−7***	***−23***
***Lung (ICD-9 162; ICD-10 C33−34)***									
Most deprived	263	250	190	168	178	150	−36	−16	−46
2	215	196	149	126	135	120	−41	−11	−48
3	185	163	130	100	100	90	−46	−10	−52
4	150	141	107	85	76	66	−44	−13	−51
Least deprived	151	114	83	68	57	51	−55	−11	−60
***All Scotland***	***191***	***172***	***131***	***106***	***106***	***90***	***−44***	***−14***	***−53***
***Prostate (ICD-9 185; ICD-10 C61)0***									
Most deprived	36	53	48	50	50	45	40	−8	28
2	45	53	52	49	47	48	8	2	10
3	43	53	56	48	50	50	12	−1	11
4	41	62	61	52	54	46	27	−14	9
Least deprived	43	54	58	55	52	46	28	−11	15
***All Scotland***	***42***	***55***	***55***	***51***	***51***	***47***	***22***	***−8***	***13***
***Colorectal (ICD-9 153, 154.0−154.1; ICD-10 C18−20)***									
Most deprived	64	60	54	50	54	49	−21	−9	−29
2	57	60	53	46	45	43	−19	−4	−22
3	57	56	51	42	42	40	−26	−6	−31
4	55	64	50	38	37	38	−30	0	−30
Least deprived	53	55	48	40	38	35	−25	−8	−31
***All Scotland***	***57***	***59***	***51***	***43***	***43***	***40***	***−24***	***−6***	***−29***
***Oesophagus (ICD-9 150; ICD-10 C15)***									
Most deprived	26	28	32	31	31	30	19	−6	12
2	18	24	27	26	29	28	49	−6	41
3	17	26	27	25	26	25	46	−2	43
4	20	24	25	25	24	22	29	−6	21
Least deprived	18	20	23	23	20	20	28	−4	23
***All Scotland***	***20***	***24***	***27***	***26***	***26***	***24***	***33***	***−6***	***25***
***Pancreas (ICD-9 157; ICD-10 C25)***									
Most deprived	21	20	18	18	18	19	−15	6	−10
2	19	20	18	20	20	19	1	−9	−8
3	22	20	15	16	17	15	−24	−10	−31
4	19	19	15	17	17	17	−14	−1	−14
Least deprived	19	15	16	16	15	16	−16	2	−14
***All Scotland***	***20***	***19***	***16***	***17***	***17***	***17***	***−14***	***−3***	***−16***
***Liver (ICD-9 155; ICD-10 C22)***									
Most deprived	7	11	11	19	19	26	188	40	304
2	5	6	10	13	14	18	161	35	253
3	7	7	9	13	14	16	82	12	104
4	4	5	7	12	11	13	183	16	229
Least deprived	5	7	6	10	9	11	114	17	150
***All Scotland***	***5***	***7***	***8***	***13***	***13***	***16***	***140***	***23***	***196***
***Bladder (ICD-9 188; ICD-10 C67)***									
Most deprived	29	23	20	20	22	17	−33	−22	−48
2	22	25	19	19	19	17	−14	−10	−23
3	22	21	19	17	16	17	−24	9	−18
4	21	21	15	15	14	14	−28	−1	−28
Least deprived	19	21	16	15	14	13	−24	−5	−28
***All Scotland***	***22***	***22***	***18***	***17***	***17***	***16***	***−25***	***−7***	***−30***
***Head and neck (ICD-9 140−149, 160−161; ICD-10 C11−14, C30−32)***									
Most deprived	18	21	23	23	24	28	30	19	55
2	15	17	17	16	17	19	9	7	17
3	13	14	13	13	13	13	5	0	5
4	10	12	10	10	9	9	−6	1	−5
Least deprived	9	9	8	8	7	8	−18	16	−5
***All Scotland***	***13***	***15***	***14***	***13***	***13***	***14***	***6***	***6***	***13***
***Stomach (ICD-9 151; ICD-10 C16)***									
Most deprived	48	42	31	18	20	19	−63	−5	−65
2	45	33	25	18	18	15	−61	−16	−67
3	40	32	22	16	15	12	−61	−22	−69
4	34	24	20	14	13	10	−59	−18	−66
Least deprived	31	22	16	12	12	8	−62	−36	−76
***All Scotland***	***39***	***31***	***22***	***15***	***15***	***12***	***−61***	***−20***	***−69***
***Kidney (ICD-9 189.0; ICD-10 C64)***									
Most deprived	10	10	11	10	11	12	−2	13	11
2	9	9	10	11	10	11	25	8	35
3	9	9	11	10	11	11	6	4	10
4	7	8	10	9	8	10	16	18	37
Least deprived	7	11	9	9	8	9	22	8	31
***All Scotland***	***8***	***9***	***10***	***9***	***9***	***10***	***13***	***9***	***23***
***Leukaemia (ICD-9 204−208; ICD-10 C91−95)***									
Most deprived	11	9	11	11	13	12	1	−1	0
2	11	8	11	12	11	11	6	−2	4
3	11	11	10	12	11	10	4	−6	−3
4	10	9	12	11	12	10	13	−21	−11
Least deprived	11	9	12	12	11	10	7	−12	−6
***All Scotland***	***11***	***9***	***11***	***12***	***12***	***10***	***7***	***−10***	***−4***

a Based on Carstairs deprivation fifths at the postcode sector level of geography.

b Based on Carstairs deprivation fifths at the data zone level of geography.

c Change between 1981 and 2016 calculated using ((1 + proportion change 1981–2011) * (1 + proportion change 2011–2016)) – 1.

**Table 2b:** Age-standardised all cancer and specific cancers mortality rates (per 100,000 population) for females, all ages, in 1981 (1980-83), 1991 (1991-93), 2001 (2000-03), 2011 (2010-13) and 2016 (2015-18) by most to least deprived fifth. Rates for all Scotland are shown in bold-italics.

	Year						% Change		
	1981^a^	1991^a^	2001^a^	2011^a^	2011^b^	2016^b^	***1981−2011***^a^	***2011−2016***^b^	***1981−2016***^c^
***All cancers (ICD-9 140−208; ICD-10 C00–C97)***									
Most deprived	343	361	350	352	366	353	3	−3	−1
2	306	321	311	302	313	303	−1	−3	−5
3	293	302	299	282	275	264	−4	−4	−7
4	285	300	275	256	241	230	−10	−4	−14
Least deprived	265	275	257	236	224	210	−11	−6	−17
***All Scotland***	***297***	***312***	***298***	***283***	***283***	***267***	***−5***	*−6*	***−10***
***Lung (ICD-9 162; ICD-10 C33−34)***									
Most deprived	72	94	99	117	127	121	63	−5	56
2	49	72	78	90	93	90	81	−3	76
3	43	58	65	72	71	69	67	−3	62
4	40	49	57	59	50	46	45	−8	33
Least deprived	37	48	46	46	39	36	24	−8	14
***All Scotland***	***48***	***64***	***69***	***75***	***75***	***69***	***56***	***−7***	***45***
***Breast (female) (ICD-9 174; ICD-10 C50)***									
Most deprived	55	55	44	39	36	36	−30	0	−30
2	53	53	45	37	39	35	−30	−11	−38
3	57	54	46	39	38	33	−31	−11	−39
4	55	57	45	37	38	33	−32	−14	−41
Least deprived	52	54	47	37	37	34	−28	−10	−35
***All Scotland***	***54***	***55***	***45***	***38***	***38***	***34***	***−30***	***−11***	***−37***
***Colorectal (ICD-9 153, 154.0−154.1; ICD-10 C18−20)***									
Most deprived	42	36	31	30	30	30	−28	−1	−28
2	44	37	29	27	27	26	−40	−2	−41
3	43	37	30	26	26	27	−40	5	−37
4	44	41	30	25	25	24	−43	−6	−46
Least deprived	42	38	30	26	24	25	−39	2	−37
***All Scotland***	***43***	***38***	***30***	***27***	***27***	***26***	***−38***	***−1***	***−39***
***Ovary (ICD-9 183.0; ICD-10 C56)***									
Most deprived	14	17	14	14	15	13	−4	−10	−13
2	14	16	18	14	14	14	−4	−4	−8
3	16	17	16	15	14	12	−4	−18	−21
4	17	16	17	15	14	14	−12	3	−9
Least deprived	15	17	17	14	14	13	−8	−8	−16
***All Scotland***	***15***	***16***	***16***	***14***	***14***	***13***	***−6***	***−8***	***−14***
***Pancreas (ICD-9 157; ICD-10 C25)***									
Most deprived	17	14	14	16	15	15	−6	1	−4
2	15	14	14	14	16	14	−8	−10	−17
3	14	13	14	13	13	13	−2	1	−1
4	14	14	10	12	12	13	−12	3	−9
Least deprived	14	11	12	11	11	11	−22	3	−19
***All Scotland***	***15***	***13***	***13***	***13***	***13***	***13***	***−10***	***−1***	***−11***
***Oesophagus (ICD-9 150; ICD-10 C15)***									
Most deprived	12	14	13	12	14	12	−2	−13	−15
2	11	14	12	11	12	11	−3	−9	−12
3	10	14	12	12	11	9	12	−17	−6
4	12	12	11	10	8	9	−14	4	−11
Least deprived	9	11	9	8	8	7	−8	−5	−12
***All Scotland***	***11***	***13***	***11***	***10***	***10***	***9***	***−4***	***−9***	***−13***
***Liver (ICD-9 155; ICD-10 C22)***									
Most deprived	3	4	6	9	9	11	178	20	234
2	3	4	4	7	6	8	137	20	185
3	2	3	4	6	6	7	203	20	264
4	2	3	4	5	5	7	147	36	235
Least deprived	2	3	4	5	4	6	158	45	274
***All Scotland***	***2***	***3***	***5***	***6***	***6***	***8***	***162***	***25***	***225***
***Non-Hodgkin lymphoma (ICD-9 200, 202; ICD-10 C82−86)***									
Most deprived	8	9	9	8	8	8	9	7	17
2	6	8	9	6	8	7	−1	−12	−13
3	5	8	9	7	7	7	56	3	60
4	6	9	7	8	7	6	32	−10	19
Least deprived	7	8	9	7	7	6	7	−19	−13
***All Scotland***	***6***	***9***	***9***	***7***	***7***	***7***	***18***	***−7***	***10***
***Corpus uteri (ICD-9 182; ICD-10 C54)***									
Most deprived	4	4	4	6	6	6	42	4	48
2	5	4	5	6	5	7	23	35	67
3	5	4	5	5	7	7	0	7	7
4	4	4	4	5	4	6	0	53	53
Least deprived	4	4	5	6	6	6	61	7	72
***All Scotland***	***4***	***4***	***5***	***5***	***5***	6	***0***	***17***	***44***
***Stomach (ICD-9 151; ICD-10 C16)***									
Most deprived	30	18	16	9	10	8	−70	−26	−77
2	23	17	12	9	8	6	−63	−26	−73
3	22	13	9	8	6	6	−66	−10	−69
4	18	14	9	5	5	5	−70	0	−70
Least deprived	16	10	7	5	6	4	−68	−24	−76
***All Scotland***	***22***	***14***	***10***	***7***	***7***	6	***−67***	***−21***	***−74***
***Leukaemia (ICD-9 204−208; ICD-10 C91−95)***									
Most deprived	6	7	6	6	6	6	13	−5	7
2	6	4	6	6	6	6	6	−6	−1
3	6	6	7	6	6	6	−6	−3	−9
4	6	5	6	6	6	5	3	−20	−17
Least deprived	7	6	6	6	5	5	−19	0	−19
***All Scotland***	***6***	***6***	***6***	***6***	***6***	6	***−3***	***−9***	***−11***
***Cervix uteri (ICD-9 180; ICD-10 C53)***									
Most deprived	13	10	6	6	6	6	−55	1	−55
2	10	10	5	4	5	5	−64	3	−63
3	8	7	5	4	4	4	−52	1	−52
4	7	6	3	3	3	2	−61	−32	−73
Least deprived	6	4	3	3	2	2	−53	−11	−58
***All Scotland***	***9***	***7***	***4***	***4***	***4***	***4***	***−57***	***−5***	***−59***

a Based on Carstairs deprivation fifths at the postcode sector level of geography.

b Based on Carstairs deprivation fifths at the data zone level of geography.

c Change between 1981 and 2016 calculated using ((1 + proportion change 1981–2011) * (1 + proportion change 2011–2016)) – 1.

Table 2a shows that between 1981 and 2011 cancer mortality rates, for males of all ages, decreased from 611 to 516 per 100,000 population in the most deprived areas and from 430 to 344 per 100,000 population in the least deprived areas; larger absolute reductions, but smaller relative reductions, in the least deprived areas. Following 2011, there were further small decreases in cancer mortality rates across all deprivation fifths. In terms of specific cancer sites, relative inequalities have widened over time for lung cancer mortality. Both absolute and relative inequalities widened for liver cancer and head and neck cancer, while absolute and relative inequalities in bladder cancer mortality narrowed over time.

Female cancer mortality rates (Table 2b) increased from 343 to 352 per 100,000 in the most deprived areas between 1981 and 2011 while declining from 265 to 236 per 100,000 population in the least deprived areas. All deprivation fifths saw a small reduction in rates following 2011. Absolute and relative inequalities widened over time. Much of this widening was driven by increasing absolute and relative inequalities in lung cancer and liver cancer mortality.

### 3.4 Contribution of specific cancers to current inequalities in cancer mortality

**Fig. 1:**
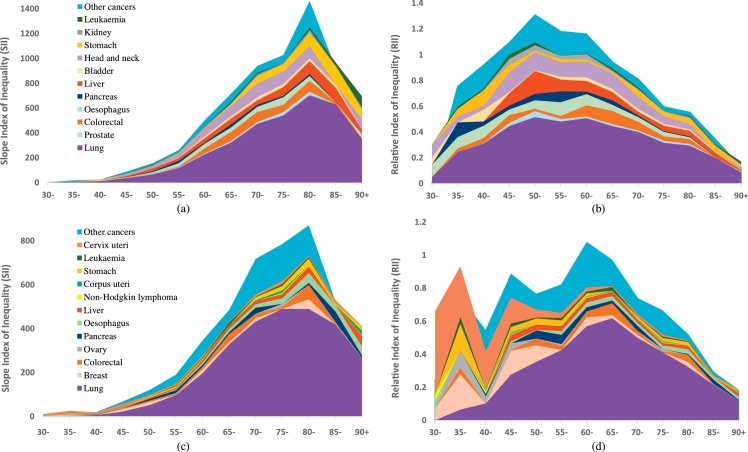
Contribution of selected specific cancers to absolute and relative inequalities in all cancers for deaths in 2016. Plots show (a) SII for males, (b) RII for males, (c) SII for females and (d) RII for females, for five-year age groups 30 to 90 + . Cancer sites are coloured consistently across males and females.

The slope index of inequality (SII) and the relative index of inequality (RII) are used to examine the contribution of specific cancers to current absolute and relative socioeconomic inequalities, respectively, in overall cancer mortality. Fig. 1(a) shows the SII in 2016, for males aged 30 to 90+. Absolute inequalities in cancer mortality gradually increase as the population ages and death rates rise. Absolute inequalities peak at around age 80–84 for males (absolute rate difference of 1461 per 100,000 between the most and least deprived populations), steadily decreasing thereafter. The width of the different bands show the extent to which absolute inequalities in cancer mortality are attributable to specific cancers. Almost half of the absolute inequalities in cancer mortality at its peak are due to inequalities in lung cancer (absolute rate difference of 702 per 100,000 population). Colorectal, liver, head and neck, and stomach cancer also contribute to absolute inequalities at the peak with smaller contributions from other cancer sites. Fig. 1(b) shows the corresponding RII plot for males aged 30 to 90+. Relative inequalities peak earlier than absolute inequalities at around age 50–54 years. The RII value at the peak is 1.2. This suggests that rates in deprived areas are around 60 % higher than the average rate (and 60 % lower than average in the least deprived areas). Together lung, liver and head and neck cancer explain just over two thirds of relative inequalities at its peak.

Fig. 1(c) shows the SII, for females aged 30–90 + . Similarly to males, absolute inequalities in cancer mortality peak at around age 80–84 for females (absolute rate difference of 871 per 100,000 between the most and least deprived populations). Again, deaths from lung cancer contributes most to absolute inequalities in female cancer rates, although the peak in lung cancer inequalities happens a little earlier at age 75−79. Relative inequalities (Fig. 1(d)) peak at around age 60–64 for females, 10 years older than for males, with a value of 1.0. This suggests that rates in deprived areas are around 50 % higher than the average rate. Relative inequalities in lung cancer mortality rates explains more than half the relative inequalities in total cancer mortality at its peak, although relative inequalities in lung cancer mortality rates don’t peak until age 65−69. At younger ages (ages 30–39), relative inequalities in total cancer mortality tend to be due to breast, ovarian and stomach cancer and cancer of the cervix.

## 4 Discussion

### 4.1 Discussion

Cancer mortality rates have declined in Scotland in the last 35 years, the gap between male and female cancer mortality rates has narrowed, and rates fell in all age groups with the exception of females aged 75 + . For males, rates of liver cancer and head and neck cancer increased overall, and did so at a faster pace in the most deprived areas while male lung cancer declined but at a faster pace in the least deprived areas. Increasing rates of liver cancer may reflect increasing levels of alcohol consumption; alcohol sales in Scotland have shown a steady increase in recent years [19]. Inequalities in liver cancer mortality may be due in part to the alcohol harm paradox, whereby at a given level of alcohol consumption, alcohol-related harms are more pronounced in those with lower socioeconomic status [20]. Alcohol is also an important risk factor for head and neck cancer. For men, a combination of alcohol and tobacco has been shown to account for a larger proportion of head and neck cancers than smoking or drinking alone [21].

For females, there was an overall increase in the rate of lung and liver cancer mortality with the increase higher in more deprived areas. Although the death rate from lung cancer remains greater for men, the narrowing of the gap between males and females reflects historical smoking patterns. Females took up smoking later than males, with the number of male smokers starting to decline around the 1950s compared to the 1970s for females. In many high income countries male lung cancer deaths are currently declining while female deaths have continued to rise [22,23]. Previous work has reported increasing lung cancer mortality rates, in over 30 countries, among females aged 50–74 [24]. We found that female lung cancer mortality rates increased between 1981 and 2011 in the over 60 s. Rates then declined between 2011 and 2016 for 60−74 year olds and remained stable for those aged 75+, which may suggest that female lung cancer mortality rates in Scotland have now peaked.

Over the last 35 years, males and females have seen large declines in deaths due to colorectal, stomach and bladder cancers with smaller declines in deaths due to pancreatic cancer and leukaemia. There have been declines in the rate of mortality of female breast cancer, ovarian cancer and cervical cancer. Small increases in mortality were observed for cancer of the uterus and prostate cancer, mainly driven by increases in the oldest age group. Liver cancer mortality rates increased for males and females. Many of these general patterns in cancer mortality have been observed in other high-income countries [[25], [26], [27]]. Despite declining overall rates, socioeconomic inequalities in cancer mortality widened over time. Males saw a slightly larger absolute reduction in cancer mortality rates in the most deprived areas compared to the least deprived, however, relative inequalities between the most and least deprived areas widened, as is often the case in the context of declining mortality rates across socioeconomic groups [28,29]. For females, both absolute and relative socioeconomic inequalities in total cancer mortality widened over the last 35 years with little change in rates in the most deprived areas and improvements in the least deprived areas. Many countries have reported widening socioeconomic inequalities in cancer mortality [[30], [31], [32]] with inequalities larger for more preventable cancer sites [33]. The World Health Organization (WHO) estimates that between 30–50 % of all cancer cases are preventable with tobacco the single greatest avoidable risk factor for cancer mortality [34]. Smoking prevalence has declined in Scotland over the last 15 years, however, in 2018 those in the most deprived areas were more likely to smoke (32 % compared to 9% in the least deprived areas) and to smoke a higher number of cigarettes (13.2 mean cigarettes in the most deprived areas compared to 9.4 in the least deprived) [35].

Current absolute socioeconomic inequalities in cancer mortality increase with age up to age 80–84 for males and females, after which they start to decrease. Relative inequalities peak earlier, at age 50–54 for males and age 60–64 for females. While absolute inequalities are often highest in age groups with most deaths, relative inequalities tend to peak earlier [36]; often due to higher premature mortality rates in the most deprived areas. Populations in the most deprived areas also experience higher levels of comorbidity and multimorbidity often with poorer access, in terms of service availability and uptake, to health services [37]. Given that absolute and relative socioeconomic inequalities in cancer mortality persist even in the oldest age groups, it is increasingly important that public health tackles socioeconomic inequalities in health, reducing inequalities across the life course in order to reduce inequalities in older age.

### 4.2 Strengths and limitations

This is a large population-wide study using high-quality population data to examine long-term trends and socioeconomic inequalities in cancer mortality rates. It is an ecological study that analyses data at an aggregate level and therefore our conclusions relate to populations and not individuals.

The choice of socioeconomic indicator is important, and findings are likely to differ across different socioeconomic measures [38]. We used Carstairs deprivation scores. As scores are census-based, they most accurately capture area deprivation around census periods 1981, 1991, 2001 and 2011. Some have questioned the current validity of the variables used in the construction of Carstairs scores [39]. Overcrowding now only affects a small percentage of the population (3.3 %) and car ownership is seen as more of a necessity in rural areas than in urban areas. Carstairs scores also only take into account male unemployment rates. However, describing inequalities over time requires a consistent socioeconomic measure and the availability of Carstairs scores over a period of four decades allows for long-term comparisons. A new small-area measure of deprivation has recently been developed for Scotland using alternative census variables including overall unemployment (male and female) and proportion of people with no school level qualifications [40], however this is only currently available for the 2001 and 2011 censuses. An alternative, widely used, deprivation measure in Scotland is the Scottish Index of Multiple Deprivation [41], although it is not recommended for examining trends pre-1996 [42].

We considered only cancer deaths here, not cancer incidence or survival, and we have no information on stage at diagnosis. Cancer incidence and survival may be subject to bias but, because death registration is a legal requirement [43], mortality data provides almost complete population coverage. We mainly focus on mortality of those aged 30+ because of the smaller numbers of death at younger ages. Around 87 % of all cancer deaths globally occur in those aged 50 and over with around 1% of all cancer deaths occurring in those aged under 15 [44]. Children in high-income countries generally have good cancer outcomes [45] while young adults (aged 15−29) tend to have more favourable outcomes relative to cancer at other ages [46].

### 4.3 Conclusions and implications

Recent public health policies in the UK have aimed to reduce socioeconomic inequalities in cancer mortality [[47], [48], [49], [50], [51]]. For these strategies to be successful it is important that they focus on upstream policies that address the socioeconomic determinants of health (i.e. tobacco control or alcohol minimum unit pricing) as they consistently achieve larger population health benefits than downstream policies [52], that target for example, individual behaviour change. Reducing cancer mortality inequalities will also require addressing the inverse care law where those from the poorest areas with worst outcomes need better access to quality healthcare services, pathways to care and cancer screening programmes. Progress in reducing socioeconomic inequalities in cancer outcomes should be monitored and regularly reported and preventative strategies should focus on long-term goals with the aim of diminishing cancer inequalities between social groups.

## Data availability

The data that support the findings of this study are available from National Records of Scotland (https://www.nrscotland.gov.uk/). Restrictions apply to the availability of these data, which were used under license for this study. Data are available from the authors with the permission of National Records of Scotland.

## Funding information

This research was funded by the 10.13039/501100007155Medical Research Council (MC_UU_00022/2) and the Scottish Government Chief Scientist Office (SPHSU17).

## Authorship contribution statement

DB wrote the manuscript with support from DIC, ADM, RD, and AHL. All authors contributed to the interpretation of the results and to the final version of the manuscript

## CRediT authorship contribution statement

**Denise Brown:** Conceptualization, Methodology, Writing - original draft, Writing - review & editing. **David I. Conway:** Conceptualization, Writing - review & editing. **Alex D. McMahon:** Conceptualization, Writing - review & editing. **Ruth Dundas:** Methodology, Writing - review & editing. **Alastair H. Leyland:** Conceptualization, Methodology, Writing - review & editing.

## Declaration of Competing Interest

The authors report no declarations of interest.
