# Supplementary material for: Cancer mortality 1981–2016 and contribution of specific cancers to current socioeconomic inequalities in all cancer mortality: A population-based study
Source: Cancer Epidemiol. 2021 Oct;74:102010. doi: 10.1016/j.canep.2021.102010 (PMC7611600; doi:10.1016/j.canep.2021.102010)
Supplement: Supplementary file 1 [file mmc1.docx]

Supplementary appendix

Table A1: Average number of cancer deaths per year in Scotland over each time period, for males and females, all ages

| Site | 1980-1983 | 1991-1993 | 2000-2003 | 2010-2013 | 2015-2018 |
| --- | --- | --- | --- | --- | --- |
| *All deaths* | *63,325* | *61,281* | *57,392* | *53,998* | *56,456* |
| All cancers | 13,923 | 15,054 | 14,986 | 15,575 | 15,945 |
| Lung | 4,073 | 4,249 | 3,930 | 4,126 | 3,997 |
| Colorectal | 1,699 | 1,730 | 1,582 | 1,551 | 1,634 |
| Prostate | 471 | 693 | 773 | 874 | 935 |
| Breast (female) | 1,213 | 1,260 | 1,117 | 1,030 | 975 |
| Oesophagus | 528 | 700 | 745 | 817 | 828 |
| Pancreas | 620 | 596 | 600 | 710 | 758 |
| Liver | 137 | 190 | 264 | 435 | 584 |
| Bladder | 453 | 489 | 439 | 480 | 496 |
| Head and neck | 281 | 355 | 359 | 418 | 478 |
| Stomach | 1,057 | 804 | 629 | 488 | 430 |
| Non-Hodgkin lymphoma | 273 | 375 | 417 | 397 | 419 |
| Kidney | 232 | 270 | 302 | 327 | 380 |
| Leukaemia | 300 | 278 | 342 | 385 | 389 |
| Ovary | 352 | 385 | 401 | 381 | 373 |
| Corpus uteri | 100 | 93 | 113 | 145 | 181 |
| Cervix uteri | 202 | 175 | 111 | 102 | 100 |

Table A2: Age-standardised mortality rates (ASMR) per 100,000 population are shown for all cancers (C00-C97) excluding non-melanoma skin cancer (NMSC; C44) and for the top ten specific cancer deaths in Scotland (PHS)^13^ and Europe (IARC)^6^ in 2018 for males and females, all ages. Other cancers reported in this paper, but which are not amongst the ten most common, are shown at the bottom of the table in italics

| **Males** | | | | **Females** | | | |
| --- | --- | --- | --- | --- | --- | --- | --- |
| **Scotland** | | **Europe** | | **Scotland** | | **Europe** | |
| *Site* | *ASMR* | *Site* | *ASMR* | *Site* | *ASMR* | *Site* | *ASMR* |
| **All cancers, excl NMSC** | **363.3** | **All cancers, excl NMSC** | **355.4** | **All cancers, excl NMSC** | **266.6** | **All cancers, excl NMSC** | **201.0** |
| Lung | 84.8 | Lung | 85.8 | Lung | 68.2 | Breast | 32.7 |
| Prostate | 43.9 | Colorectal | 43.8 | Breast | 33.9 | Lung | 28.9 |
| Colorectal | 41.4 | Prostate | 38.9 | Colorectal | 27.1 | Colorectal | 26.1 |
| Oesophagus | 24.8 | Pancreas | 21.1 | Ovary | 13.3 | Pancreas | 14.8 |
| Pancreas | 18.1 | Stomach | 20.1 | Pancreas | 13.2 | Ovary | 10.7 |
| Liver | 16.3 | Bladder | 17.4 | Oesophagus | 9.7 | Stomach | 9.4 |
| Bladder | 14.3 | Liver | 16.4 | Non-Hodgkin lymphoma | 6.7 | Corpus uteri | 7.0 |
| Head and neck | 14.0 | Kidney | 11.5 | Liver | 6.6 | Leukaemia | 6.4 |
| Stomach | 11.9 | Leukaemia | 11.5 | Corpus uteri | 6.5 | Cervix uteri | 6.3 |
| Kidney | 10.4 | Oesophagus | 11.0 | Stomach | 6.1 | Liver | 6.3 |
|  |  |  |  |  |  |  |  |
| *Leukaemia* | *10.1* | *Non-Hodgkin lymphoma* | *8.8* | *Bladder* | *6.0* | *Non-Hodgkin lymphoma* | *5.1* |
| *Non-Hodgkin lymphoma* | *9.6* | *Head and neck^1^* |  | *Leukaemia* | *5.5* | *Kidney* | *4.6* |
|  |  |  |  | *Kidney* | *5.2* | *Bladder* | *3.5* |
|  |  |  |  | *Head and neck* | *4.9* | *Oesophagus* | *2.4* |
|  |  |  |  | *Cervix uteri* | *2.8* | *Head and neck^1^* |  |

^1^ Head and neck (C00-14, C30-32) cancers are not combined in ECIS reporting. Head and neck cancer sites are broken down by Lip, oral cavity (C00-06), Salivary glands (C07-08), Oropharynx (C09-10), Nasopharynx (C11), Hypopharynx (C12-13) and Larynx (C32). ASMR’s for males are 5.5 (Larynx), 5.3 (Lip, oral cavity), 3.2 (Oropharynx), 2.5 (Hypopharynx), 0.9 (Salivary glands) and 0.6 (Nasopharynx). ASMR’s for females are 1.5 (Lip, oral cavity), 0.6 (Oropharynx), 0.5 (Larynx), 0.3 (Salivary glands), 0.2 (Hypopharynx) and 0.2 (Nasopharynx)

Table A3: All cancers, excluding non-melanoma skin cancer (NMSC)

|  | Males | | | | | |  | Females | | | | | |
| --- | --- | --- | --- | --- | --- | --- | --- | --- | --- | --- | --- | --- | --- |
|  | Years | | | | | *% Change* |  | Years | | | | | *% Change* |
| Age | 1981 | 1991 | 2001 | 2011 | 2016 | ***1981 - 2016*** |  | 1981 | 1991 | 2001 | 2011 | 2016 | ***1981 - 2016*** |
| ***All cancers, excl NMSC (ICD-9 140-208, excl 173; ICD-10 C00-C97, excl C44)*** | | | | | | | | | | | | | |
| 0-14 | 5 | 3 | 4 | 2 | 2 | *-36* |  | 4 | 4 | 2 | 2 | 2 | *-* |
| 15-29 | 9 | 6 | 6 | 5 | 4 | *-51* |  | 8 | 6 | 5 | 5 | 4 | *-45* |
| 30-44 | 37 | 31 | 24 | 21 | 22 | *-42* |  | 53 | 43 | 33 | 30 | 29 | *-46* |
| 45-59 | 279 | 256 | 206 | 159 | 147 | *-47* |  | 252 | 236 | 194 | 163 | 147 | *-41* |
| 60-74 | 1178 | 1195 | 1026 | 835 | 762 | *-35* |  | 677 | 745 | 674 | 619 | 562 | *-17* |
| 75+ | 2581 | 2778 | 2671 | 2539 | 2378 | *-8* |  | 1330 | 1435 | 1535 | 1544 | 1515 | *14* |
| ***All ages*** | ***494*** | ***508*** | ***458*** | ***404*** | ***376*** | ***-24*** |  | ***296*** | ***311*** | ***297*** | ***282*** | ***266*** | ***-10*** |

Cancer mortality rates are rounded to the nearest whole number while % change shows the percentage change in actual (unrounded) rates. Note that % change is not calculated, for a particular age group, when rates are consistently <5 per 100,000 population over time.

| 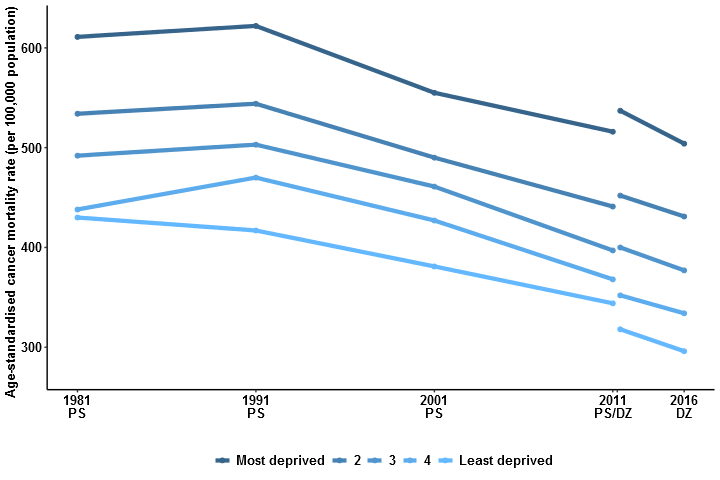 | 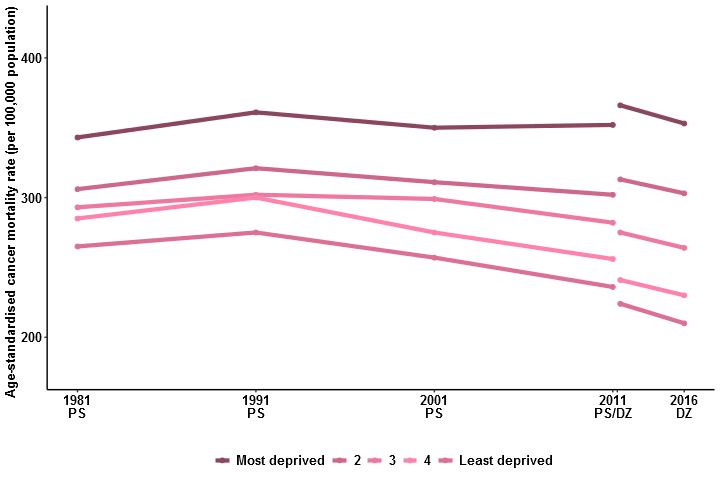 | |
| --- | --- | --- |
| (a) | | (b) |

**Figure A1:** All cancer mortality, by deprivation fifths, for deaths 1981, 1991, 2001 and 2011 (postcode sectors; PS) and deaths 2011 and 2016 (data zones; DZ) for (a) Males, all ages and (b) Females, all ages
